# Occurrence of urogenital mycoplasmas in men with the common genitourinary diseases

**DOI:** 10.1007/s42770-021-00620-1

**Published:** 2021-09-24

**Authors:** Dominika Smolec, Alicja Ekiel, Piotr Kłuciński, Jan Kawecki

**Affiliations:** 1 Department of Medical Microbiology, Faculty of Medical Sciences in Katowice, Medical University of Silesia, Medyków 18 Street, 40-752 Katowice, Poland; 2 Med Holding Emil Michalowski Specialist Hospital, Katowice, Poland

**Keywords:** *Mycoplasma*, *Ureaplasma*, Genitourinary tract carcinoma, Urolithiasis, Benign prostatic hyperplasia

## Abstract

Many serious and fatal infections with urogenital mycoplasmas in immunocompromised patients have been reported. *M. genitalium* is recognized as a cause of male urethritis and other common genitourinary diseases. The aim of the study was to estimate prevalence of urogenital mycoplasmas which can cause complications in men with common genitourinary diseases. Study included 85 men with genitourinary tract carcinoma (*n* = 35), urolithiasis (*n* = 36), and BPH (benign prostatic hyperplasia) (*n* = 14). The control group consisted of 50 healthy men. FVU (first void urine) samples were examined by PCR for the presence of urogenital mycoplasmas DNA. Occurrence of urogenital mycoplasmas was significantly more common in study group compared with control 24/85 (28.2%) and 7/50 (14%), respectively (*p* = 0.05). In men with urolithiasis, positive results for mycoplasmas DNA were significantly more frequent than in control: 33.3% vs. 14% (*p* < 0.05). In patients with urolithiasis DNA of *U. urealyticum* was most often found, while in the genitourinary carcinoma and BPH groups, *U. parvum* was more frequent. Incidence of *M. fermentans* was also significantly higher in the urolithiasis group vs. control (*p* = 0.03). A higher percentage of positive results for urogenital mycoplasma DNA in study group has been found. Further studies are required to confirm the role of urogenital mycoplasmas in the development of infectious complications among patients with urolithiasis, genitourinary carcinoma, and BPH.

## Introduction

Urogenital mycoplasmas are commonly found as a part of the normal microbiome of the human urogenital tract. Some of them, mainly *Mycoplasmoides genitalium* (previous *Mycoplasma genitalium*) [1], are recognized as a cause of urethritis and risk factor of developing prostatitis, epididymitis, cervicitis, pelvic inflammatory disease (PID), and bacterial vaginosis (BV); they can have a negative impact on fertility and may be cause of pathological course of pregnancy, as well as low birth weight of the newborn [2]. Urogenital mycoplasmas may be also an etiological factor of opportunistic infections in patients with genitourinary system and other diseases, especially in patients with a decreased immunity resulting from the underlying disease or ongoing treatment. In patients treated with peritoneal dialysis, hyperammonemia, periaortic abscess following heart–lung transplantation, peritonitis *Ureaplasma urealyticum*, and *Metamycoplasma hominis* (previous *Mycoplasma hominis*) [1] mainly are among the etiological agents of infections [3–5]. Other species, such as *Mycoplasmopsis fermentans*, *Malacoplasma penetrans*, and *Mycoplasmoides pirum* (previous *Mycoplasma fermentans*, *Mycoplasma penetrans*, *Mycoplasma pirum*) [1], are rarely considered in the studies of human biological materials.

Our study aimed to estimate prevalence of urogenital mycoplasmas (*Mycoplasma* spp. and *Ureaplasma* spp.) due to the possibility of complications in patients with genitourinary cancer, urolithiasis, and benign prostatic hyperplasia (BPH) compared to control group. To our knowledge, this is the first type study in Poland.

## Patients and methods

### Patients

The study included 85 men with genitourinary tract diseases. All patients were under care of Med Holding Emil Michalowski Specialist Hospital (urology hospital in the southern Poland). Three groups of men were distinguished. First, patients with genitourinary cancer (prostate cancer, bladder cancer, and kidney cancer), (*n* = 35, mean age 67 ± 9.0) were at the diagnostic stage before any specialized oncological treatment. Second group, patients with urolithiasis (*n* = 36, mean age 49 ± 12.2), and the third group, men with BPH (*n* = 14, mean age 65 ± 7.0). All 85 men were screened for the past history of diseases and laboratory test: morphology (blood cell count) and general urinalysis. The characteristics of patients in study groups are presented in the table (Table 1).

**Table 1:** The characteristics of patients

	Genitourinary cancer (*n* = 35)	Urolithiasis (*n* = 36)	BPH (*n* = 14)	Total (*n* = 85)
Age (years)	67 ± 9.0	49 ± 12.2	65 ± 7.0	47 ± 16.1
Selected laboratory parameters				
Urine				
Leukocyturia > 5	10 (4)	23 (14)	4 (0)	37 (18)
Hematuria [> 3 RBC]	9 (4)	20 (11)	3 (0)	32 (15)
Blood				
Leukocytosis *N*: > 11000/µL	3 (0)	5 (1)	0	8 (1)
PSA > 4 ng/ml	12 (5)	0	6 (2)	18 (7)
Other diseases				
Hypertension	19 (9)	13 (5)	7 (2)	39 (16)
Cardiological diseases	9 (0)	2 (0)	2 (0)	13 (0)
Smoking	3 (1)	0	0	3 (1)
Type 2 diabetes	2 (0)	0	2 (0)	4 (0)

The results show arithmetic mean ± SD or number (%) of patients with positive results for mycoplasma detection

*Total*, patients with the following: genitourinary cancer, urolithiasis, BPH

*BPH*, benign prostatic hyperplasia

*PSA*, prostate-specific antigen

The control group consisted of sexually active, healthy men (*n* = 50, mean age 47 ± 16.1) without subjectively experienced symptoms from the urogenital tract. All included men gave informed consent for the study.

This study was approved by Bioethical Commission of the Medical University of Silesia in Katowice (KNW/0022/KB1/48/14, KNW/022/KB1/48/I/14/16, KNW/0022/KB1/48/II/14/16/17). Exclusion criteria of patients were based on lack of consent, antibiotic therapy and/or chemotherapy and antifungals (at least 4 weeks before examination), diagnosed STI, and the catheter and endoscopic surgery in the urogenital tract (at least 4 weeks before examination).

### Specimens

Patients were informed about the urine collection instructions. Samples of morning FVU (*first void urine*) were collected (5–10 mL) in sterile plastic container and transported at + 4 °C to the Department of Medical Microbiology Medical University of Silesia in Katowice, Poland.

### Methods

DNA extraction was done from the pellet obtained after centrifugation (15 000 g, 30 min, at 4 °C) of 4 ml FVU using Gene MATRIX, Bacterial & Yeast Genomic DNA Purification Kit (EURx). Species identification was performed by the polymerase chain reaction (PCR) using specific primers for *U. urealyticum*, *U. parvum*, *M. hominis*, *M. fermentans, *and *M. pirum* (Table 2) [6–8]. Amplifications were conducted using *Taq* PCR Core Kit (Qiagen Inc.) in thermocycler Mastecycler (Eppendorf AG). Negative samples were checked for presence of amplification inhibitors by PCR reactions with beta-globin control primers. Amplified products were visualized under UV light after electrophoresis in 2% agarose gel, containing ethidium bromide and recorded in the system for image archiving and analysis (GeneSys, Syngene). Examination of *M. genitalium* DNA was performed by real-time PCR using the RealBest DNA *Chlamydia trachomatis/Mycoplasma genitalium* test (Vector-Best, Russia) according to the manufacturer’s instruction. Reference strains *U. urealyticum* ATCC 27,618, *U. parvum* ATCC 27,815, and genomic DNA (ATCC33530D) of *M. genitalium* ATCC 33,530, *M.* *fermentans* ATCC 199989D, and *M. pirum* ATCC 25960D were used as positive controls*.*

**Table 2:** PCR conditions and the primer sequences used for the detection of DNA *U. parvum*, *U. urealyticum*, *M. hominis*, *M. pirum*, and *M. fermentans*

Species primers	Oligonucleotide sequence 5′–3′	Product size (bp)	Reference
*U. parvum* UMS-57 UMA-222	5′ – YAA ATC TTA GTG TTC ATA TTT TTT AC – 3′ 5′ – GTA AGT GCA GCA TTA AAT TCA ATG– 3′	326	[5]
*U. urealyticum* UMS-170 UMA-263	5′ – GTA TTT GCA ATC TTT ATA TGT TTT CG– 3′ 5′ – TTT GTT GTT GCG TTT TCT G– 3′	476	[5]
*M. hominis* MHF MHR	5′- ATA CAT CGA TGT CGA GCG AG—3′ 5′- CAT CTT TTA GTG GCG CCT TAC -3′	270	[6]
*M. pirum* primer 7 primer 8	5′ – ATA CAT GCA AGT CGA TCG GA – 3′ 5′ – ACC CTC ATC CTA TAG CGG TC – 3′	180	[7]
*M.* *fermentans* RW004 RW005	5′ – GGA CTA TTG TCT AAA CAA TTT CCC – 3′ 5′ – GGT TAT TCG ATT TCT AAA TCG CCT – 3′	206	[7]

PCR conditions:

*U. parvum*: 94˚C/3 min, 35 × (95˚C/30 s, 58˚C/30 s, 72˚C/1 min), 72˚C/7 min

*U. urealyticum*: 94˚C/3 min, 35 × (95˚C/30 s, 55˚C/30 s, 72˚C/1 min), 72˚C/7 min

*M. hominis*: 95˚C/10 min, 35 × (94˚C/40 s, 58˚C/40 s, 72˚C/40 s), 72˚C/15 min

*M. pirum*: 94˚C/2 min, 35 × (94˚C/30 s, 55˚C/30 s, 72˚C/1 min), 72˚C/5 min

*M. fermentans*: 94˚C/2 min, 35 × (94˚C/30 s, 55˚C/45 s, 72˚C/50 s), 72˚C/5 min

### Statistical analysis

Statistical analysis was performed in the Dell Statistica Version 13 (Dell INC. [2016] software.dell.com). Intergroup differences and age structure were analyzed using the chi-square test. *p*-values below 0.05 were considered as statistically significant.

## Results

**Fig. 1:**
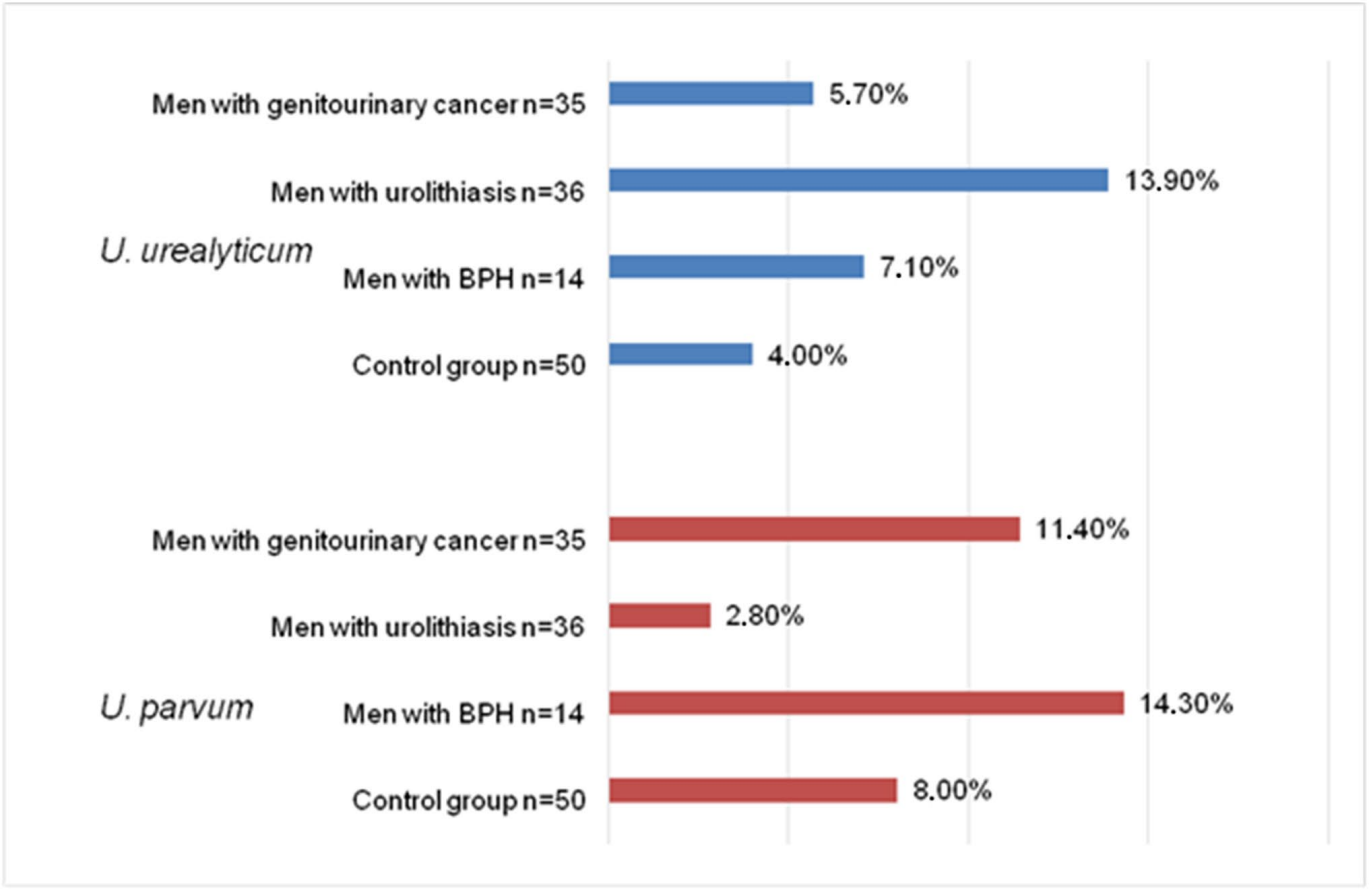
Prevalence of *Ureaplasma* species in study groups

The prevalence of urogenital mycoplasmas was found more often in men of the study group than in the control (28.2% and 14% respectively, *p* = 0.05). Occurrence of urogenital mycoplasmas in the group of patients with urolithiasis (33.3%) compared to control (14%) has shown statistically significant difference (*p* = 0.03). Also in this study group, DNA of *U. urealyticum* was most frequently found, while in the remaining groups, *U. parvum* were more often observed (Fig. 1). In other groups, positive samples for *Mycoplasma* or *Ureaplasma* DNAs were also more often detected compared to control; however, the differences were not statistically significant (Table 3).

**Table 3:** Frequency of urogenital mycoplasmas

	Genitourinary cancer (*n* = 35)	Urolithiasis (*n* = 36)	BPH (*n* = 14)	Total (*n* = 85)	Control group (*n* = 50)
	*N* (%)				
*U. urealyticum*	2 (5.7)	3 (8.3)	1 (7.1)	6 (7.1)	1 (2)
*U. parvum*	4 (11.4)	1 (2.8)	2 (14.3)	7 (8.2)	3 (6)
*M. hominis*	1 (2.9)	3 (8.3)	0	4 (4.7)	1 (2)
*M. pirum*	0	1 (2.8)	0	1 (1.2)	1 (2)
*M. fermentans*	2 (5.7)	2 (5.5)	0	4 (4.7)	0
*U. urealyticum* + *M. hominis*	0	1 (2.8)	0	1 (1.2)	0
*U. urealyticum* + *M. fermentans*	0	1 (2.8)	0	1 (1.2)	0
*U. urealyticum* + *U. parvum* + *M. hominis*	0	0	0	0	1 (2)
	9 (25.7)	12 (33.3) #*p* = 0.03	3 (21.4)	24 (28.2) *p = 0.05	7 (14)

*p* values < 0.05 are considered as statistically significant

*Total*, patients with the following: genitourinary cancer, urolithiasis, BPH

*BPH*, benign prostatic hyperplasia

*Total vs. control group, #Urolithiasis vs. control group

More than one *Mycoplasma* spp. was found in the same patient. *U. parvum* DNA occurred more frequently than other mycoplasmas. *M. pirum* and *M. fermentans* occurred with a low frequency only in the study groups; however, incidence of *M. fermentans* DNA was significantly higher in the urolithiasis group vs. controls (*p* = 0.03). In both groups, men with genitourinary tract diseases and control no DNA of the *M. genitalium* was found.

No relationship was found between the presence of urogenital mycoplasmas and the accompanying diseases or the results of laboratory tests in the study group. Age analysis had shown that positive results were more common in 21–30-year-old men compared to others (*p* = 0.05).

## Discussion

The presence of mycoplasmas in the urogenital tract of women and men and the potential association of development of diseases has been studied for many years. The introduction of molecular biology methods and FVU as a diagnostic material increased the frequency of tests in men. *Ureaplasma* and *Mycoplasma* DNA occurs in a few percent of men without any symptoms of infection. The prevalence of *U. urealyticum* and *M.* *hominis* compared to control group in our study was reported: 9.4% (8/85) vs 4% (2/50) and 5.8% (5/85) vs 4% (2/50) respectively. Positive results for *U. urealyticum* in control groups performed by other authors were 2.5–8.0% [8–11]; and for *M. hominis*, 1–6% [11–16]. Most of these papers were based on infertility study and used sperm as a material for the investigation (not FVU), although the data performed by Gdoura et al. have shown that the results obtained from sperm and FVU were similar [17].

Among patients with urolithiasis occurrence of urogenital mycoplasmas was significantly more common compared to control group – 33.3% vs. 14% (*p* < 0.05); *U.* *urealyticum* in this group was the most frequent. Only in urolithiasis group, *U. urealyticum* occurred more often than *U. parvum*. In published studies, *U.* *urealyticum* are more likely to cause symptomatic infections than *U. parvum*. *Ureaplasma* spp. may affect the formation of urinary stones leading to recurrent urolithiasis in patients by the creation of urease. In sterile normal urine, urease is not present; therefore, the basic condition for the formation of struvite stones in the urinary tract is the presence of urease-producing bacteria such as *U. urealyticum*. Under the influence of the urease produced, the pH of the urine changes to create a stone-friendly environment [18, 19]. The role of *Ureaplasma* spp. in the production of urinary tract stones was also demonstrated in vivo in rats [20].

Molecular tests implemented for routine diagnostics in addition to species detected by microculture methods (*Ureaplasma* spp. and *M. hominis*) usually also include detection of *M. genitalium*. Occurrence of *M.* *genitalium* infection is 1–3% in men, according to community-based studies from the USA, UK, Scandinavia, and Australia [21–24]. In Miyake et al.’s study, the positive rate of *M. genitalium* in the group of man with human prostate cancer and BPH was very high 45.5% and 33.18% respectively [25]. However, the lack of positive results for *M. genitalium* in our study is not surprising for the group of patients without symptoms and inflammatory features of the genitourinary tract. Similar results were also confirmed by other authors. The low prevalence of *M. genitalium* in samples from infertile men and healthy men in control was also reported by Plecko et al. [26].

In our groups with benign prostatic hyperplasia and genitourinary cancer, the frequency of urogenital mycoplasmas detection was 21.4% and 25.7%, respectively (in control — only 14%). Miyake et al. in the group of man with human prostate cancer and BPH did not show the presence of *U. urealyticum* in examining surgical and biopsy specimens [27]. In these groups, infections are considered as a factor influencing on inflammation, progression of symptoms, or factors complicating the diagnostic or therapeutic process [27, 28].

Miyake et al. in the study included testing for the presence of *M. hyorhinis* DNA, although did not obtain positive results testing surgical specimens from man with prostate cancer and BPH [25]. Studies of *M. pirum*, *M. penetrans*, and *M. fermentans* (included our study) are rarely done, but can detected in patients with malignances and other genitourinary system diseases; however, further research is needed to clarify role of these microorganisms in etiology of mentioned diseases [29–31]. When there are difficulties in detecting the etiological agent in samples collected from patients with symptoms of infection, the presence of mentioned mycoplasmas is most often suspected; in such situation, extending the research on mycoplasmas may be positive.

The limitations of this study were as follows: small number of patients in groups and it is impossible to generalize our results; real-time PCR, which possessed higher sensitivity, was used only for detection of *M. genitalium* DNA. For other mycoplasmas, we used conventional PCR because real-time PCR tests, especially for *M. pirum* and *M. fermentans*, were not available, when this study was designed.

## Conclusions

Higher percentage of urogenital mycoplasmas DNA in study group compared with control has been found in our study. In men with urolithiasis, DNA of urogenital mycoplasmas was significantly more frequent than in controls and *U. urealyticum* was most often detected, while in the remaining groups, *U. parvum* was most frequently observed. Incidence of *M. fermentans* was significantly higher in the urolithiasis group vs. controls.

It is important to consider urogenital mycoplasmas as a potential etiology of urogenital infection when clinical symptoms are present but etiology is unknown or uncertain.

## Data Availability

All data generated or analyzed during this study are included in this published article (and its supplementary information files).
